# A Law to Incorporate Dental Societies for the Purpose of Improving and Regulating the Practice of Dentistry in the State of New York

**Published:** 1868-06

**Authors:** 


### A LAW TO INCORPORATE DENTAL SOCIETIES FOR THE PURPOSE OF IMPROVING AND REGULATING THE PRACTICE OF DENTISTRY IN THE STATE OF NEW YORK.

*The People of the State of New York, represented in Senate and Assembly, do enact as follows:*

SECTION 1. It shall be lawful for the dentists in the several judicial districts of the supreme court of this State to meet together at the following named places, to-wit: In district one, at the Cooper Institute, in the city of New York; district two, at ——————, in the city of Brooklyn; district three, at the Delaven House, in the city of Albany; district four, at the Clarendon Hotel, Saratoga Springs; district five, at Stanwix Hall Hotel, in the village of Rome; district six, at the Lewis House, in the village of Binghampton; district seven at the Canandaigua Hotel, in the Village of Canandaigua; district eight, at Medical Hall, in the city of Buffalo, on the first Tuesday of June, eighteen hundred and sixty-eight, at two o'clock in the afternoon of that day, and such dentists so convened as aforesaid, or any part of them, not less than fifteen in number, shall proceed to the choice of a president, vice-president, secretary, and treasurer, who shall hold their offices for one year, and until others shall be chosen in their places; and whenever said societies shall be organized as aforesaid, they are hereby constituted bodies corporate, in fact and under the names of "The District Dental Society" of the respective judicial districts where they shall be located: provided always, that if the dentists residing in any district shall not meet and organize themselves as aforesaid, it shall be lawful for them, at the call of fifteen dentists residing in any such district, to meet at such other time and place as they shall designate; and their proceedings shall be as valid as if such meeting had been at the time before specified.

SEC. 2. Each of said district societies when organized as aforesaid, shall elect eight delegates, who shall meet at the capitol, in the city of Albany, on the last Tuesday of June, eighteen hundred and sixty-eight, and proceed to organize a State dental society, which shall be named "The Dental Society of the State of New York," and, being met, not less than thirty-three in number, shall proceed to elect, and shall thereafter annually elect, a president, vice-president, secretary and treasurer, who shall hold their offices for one year, and until others shall be chosen in their places; and said society shall be a body corporate, under the name and style aforesaid.

SEC. 3. The secretaries of each of the district societies shall lodge, in the county clerk's office of some county within their district; a copy of all the proceedings and records of their organization; and it shall also be the duty of the secretary of the State Dental Society, in like manner, to lodge, in the office of the secretary of State, a copy of its records and proceedings had at the organization thereof; and the said county clerks, respectively, and the secretary of State shall file the same in their respective offices, and shall receive therefor a fee of

SEC. 4. At the first meeting of said State Dental Society, the same being duly organized as aforesaid, the delegation from each district society shall be divided into four classes of two delegates each, who shall serve one, two, three and four years respectively, and until others shall be elected in their places; and the said district societies, at each annual meeting thereafter, shall choose two delegates to the State society, to serve each four years, and fill all vacancies in their respective delegations that may have occurred by death or otherwise.

SEC. 5. Each of the incorporated dental colleges of this State may annually elect two delegates to the State Dental Society, who shall be entitled to all the privileges, and subject to the same rules and regulations as other delegates.

SEC. 6. The said State Dental Society may elect permanent members of said society from among eminent dentists residing in this State, but not to exceed twenty in number, at its first meeting, nor more than five in any one year thereafter ; which members so elected shall be entitled to all the privileges of delegate members, but shall receive no compensation for their attendance on meetings of the State society, except when sent as delegates by the district societies or colleges aforesaid. And the said State society may elect honorary members from any State or country; but no person shall be elected an honorary member who is eligible to regular membership, nor shall any honorary member be entitled to vote or hold any office in said society.

SEC. 7. The several district societies established as aforesaid, at their annual meetings, shall appoint not less than three nor more than five censors, to continue in office for one year, and until others are chosen, who shall constitute a district board of censors, whose duty it shall be carefully and impartially to inquire into the qualifications of all persons who shall present themselves, within the districts where they reside, for examination, and report their opinion, in writing, to the president of said district society, who shall thereupon issue, on the recommendation of said board of censors, a certificate of qualification to such person or persons, countersigned by the secretary, and bearing the seal of the said district society.

SEC. 8. The State Dental Society, organized as aforesaid, at its first meeting shall appoint eight censors, one from each of the said district societies, who shall constitute a State board of censors, and at the first meeting of said board the members shall be divided into four classes, to serve one two, three and four years respectively, and said State Dental Society shall, at each annual meeting thereafter, appoint two censors, to serve each four years and until their successors shall be chosen, and fill all vacancies that may have occurred in the board by death, or otherwise. Each district society shall be entitled to one and only one member of said board of censors. Said board of censors shall meet at least once in each year, at such time and place as they shall designate, and being thus met they, or a majority of them, shall carefully and impartially examine all persons who are entitled to examination under the provisions of this act, and who shall present themselves for that purpose, and report their opinion in writing to the president of said State Dental Society, and on the recommendation of said board it shall be the duty of the president, aforesaid, to issue a diploma to such person or persons, countersigned by the secretary, and bearing the seal of said society.

SEC. 9. All dentists in regular practice at the time of the passage of this act, and all persons who shall have received a diploma from any dental college in this State, and all students who shall have studied and practiced dental Surgery with some accredited dentist or dentists for the term of four years, shall be entitled to an examination by said board of censors. Deductions from such term of four years shall be made in either of the following cases:

1. If the student, after the age of sixteen, shall have pursued any of the studies usual in the colleges of this State, the period, not exceeding one year, during which he shall have pursued such studies shall be deducted.

2. If the student, after the age of sixteen, shall have attended the complete course of lectures of any incorporated dental college in this State, or elsewhere, one year shall be deducted.

SEC. 10. Every person on receiving a diploma from the State Dental Society shall pay into the treasury thereof the sum of twenty dollars, and on receiving a certificate of qualification from the dental society of any district the sum of twenty dollars into the treasury thereof.

SEC. 11. The dental societies of the respective districts, and the dental society of the State, may purchase and hold such real and personal estate as the purposes of their respective corporations may require. The district societies each not exceeding in value the sum of five thousand dollars, and the State Dental Society not exceeding twenty-five thousand dollars in value.

SEC. 12. The respective societies herein provided for may make all needful by-laws, rules and regulations, not inconsistent with any existing law, for the management of the affairs and property of said societies respectively, and providing for the admission and expulsion of members, provided that such by-laws, rules and regulations of the respective district societies shall not be repugnant to nor inconsistent with the by-laws, rules and regulations of the State Dental Society.

SEC. 13. All dentists who shall have been in regular practice in this State, at the time of the passage of this act, and all persons who shall have received a certificate of qualification from any district society, shall be eligible to membership in said district societies.

SEC. 14. The dental society of the State of New York shall be entitled to all the privileges and immunities granted to the medical societies of this State.

SEC. 15. This act shall take effect immediately.

